# Expression of a novel CNPY2 isoform in colorectal cancer and its association with oncologic prognosis

**DOI:** 10.18632/aging.101324

**Published:** 2017-11-13

**Authors:** Jianhong Peng, Qingjian Ou, Jian Guo, Zhizhong Pan, Rongxin Zhang, Xiaojun Wu, Yujie Zhao, Yuxiang Deng, Caixia Li, Fulong Wang, Liren Li, Gong Chen, Zhenhai Lu, Peirong Ding, Desen Wan, Yujing Fang

**Affiliations:** ^1^ Department of Colorectal Surgery, Sun Yat-sen University Cancer Center, State Key Laboratory of Oncology in South China, Collaborative Innovation Center for Cancer Medicine, Guangzhou, Guangdong 510060, P. R. China; ^2^ Department of Experimental Research, Sun Yat-sen University Cancer Center; State Key Laboratory of Oncology in South China, Collaborative Innovation Center for Cancer Medicine, Guangzhou, Guangdong 510060, P. R. China; ^3^ Senboll Biotechnology Co., Ltd., Pingshan Bio-pharmacy Business Accelerator Unit 205, Shenzhen, Guangdong 518000, P. R. China; ^4^ School of Mathematics and Computational Science, Sun Yat-sen University, Guangzhou, Guangdong P. R. China

**Keywords:** CNPY2 isoform2, colorectal cancer, expression, prognosis

## Abstract

Colorectal cancer (CRC) is a leading cause of cancer-related mortality. Recently, we identified a novel biomarker, canopy fibroblast growth factor signaling regulator 2 (CNPY2) isoform2, and subsequently investigated its expression and prognostic value in CRC patients. We initially generated CNPY2 isoform2 monoclonal antibodies and examined CNPY2 isoform2 expression in CRC cell lines and tissues using quantitative real-time polymerase chain reaction, western blot and immunohistochemistry analyses. We found that CNPY2 isoform2 expression significantly increased in tumor cell lines and tissues compared with that in normal colon epithelial cells and tumor-adjacent normal tissues. Survival analysis indicated that patients with low CNPY2 isoform2 expression had poorer 5-year overall survival (OS) in both the training cohort (41.7% vs. 77.7%, *P* = 0.007) and validation cohort (47.1% vs. 78.8%, *P* = 0.002). In multivariable analysis, CNPY2 isoform2 was identified as a predictor of 5-year OS in both the training cohort [hazard ratio (HR) = 5.001; 95% confidence interval (CI) 2.156–11.598, *P* < 0.001) and validation cohort (HR= 2.443; 95% CI 1.197- 4.983, *P* = 0.014). In conclusion, CNPY2 isoform2 represents as a novel and valuable prognostic indicator for CRC patients, while the oncologic function of CNPY2 requires further study.

## INTRODUCTION

Colorectal cancer (CRC) has been ranked as the third leading cause of cancer-related deaths in China, with an estimated 191,000 deaths in 2015 [1]. Development of distantly metastatic disease is the major cause of death regardless of effective surgical procedures and systematic chemotherapy. Approximately 20% to 25% of patients were initially diagnosed as having synchronous metastases, and approximately half of the cases ultimately developed metachronous disease after primary tumor resection [2,3]. Recent genetic and molecular analyses of CRC identified a set of prog-nostic and predictive biomarkers, including RAS status, BRAF mutation and mismatch repair protein (MMR) expression, aiding identification of patients at a higher risk of disease recurrence or progression [4-6]. Although available biomarkers are commonly adopted in predicting long-term outcome, we previously observ-ed that a proportion of patients were misjudged [7,8]. Therefore, identifying novel markers to screen out various prognostic risk subgroups to guide individual treatment is urgently needed.

Canopy fibroblast growth factor signaling regulator 2 (CNPY2) belongs to the canopy family of proteins (which includes CNPY1–4), containing a saposin B-type domain and an endoplasmic reticulum (ER) retention sequence (HDEL)[9]. There are two CNPY2 variants encoding two different CNPY2 isoforms. Transcript 1 encodes the longer isoform (20.65 kDa), named isoform1. Compared to transcript 1, transcript 2 lacks several 3′ exons but has an alternate 3′ segment and encodes the shorter isoform (9.12 kDa), named isoform2 [10]. Both isoforms have a homogeneous region at the N-terminal but possess different C-terminals (Supplemental Figure 1). Previous studies have shown that CNPY2 isoform1 was widely expressed in multiple organs and tissues and was further identified as a secreted angiogenic growth factor, promoting smooth muscle cell migration, proliferation, and tissue revascularization in vivo [11,12]. However, the biological function of CNPY2 isoform 2 is still unclear. In addition, data on human tissue and cell populations that express this isoform2 transcript in vivo are also lacking. Thus, CNPY2 isoform2 requires further investigation as a novel biomarker in oncologic research to determine its potential clinical value.

In the current study, we first examined the expression of CNPY2 isoform2 in CRC cell lines and tissue as well as normal colonic epithelial cells and tumor-adjacent normal tissues. Furthermore, we explored the relation-ship between its expression and oncologic prognosis to determine whether CNPY2 can serve as a valuable prognostic predictor for CRC patients.

## RESULTS

### CNPY2 isoform expression in CRC cell lines and tissues

**Figure 1:**
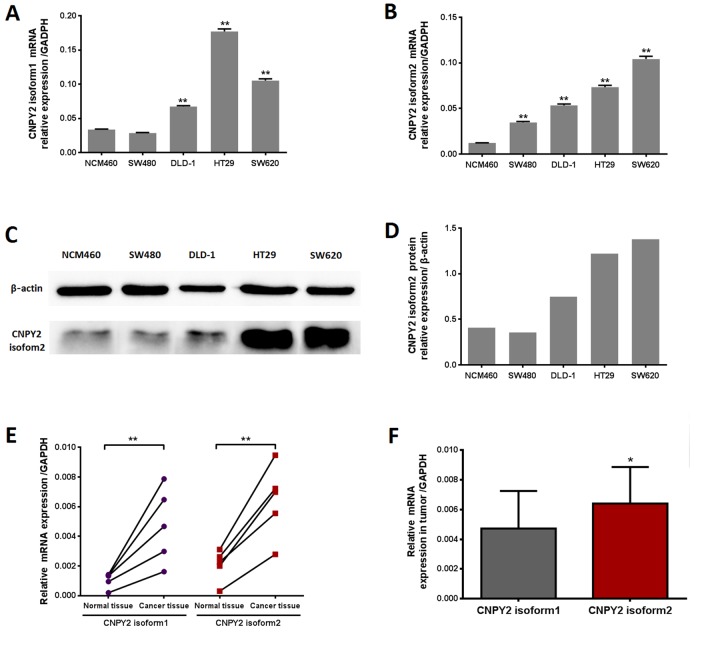
CNPY2 mRNA expression evaluated by RT-PCR (**A**) CNPY2 isoform1 mRNA was significantly higher in CRC cell lines (DLD-1, SW620, and HT29) than normal colonic epithelial cells (NCM460) **, *P* < 0.01. (**B**) CNPY2 isoform2 mRNA was significantly increased in all CRC cell lines (SW480, DLD-1, SW620, and HT29) compared to that in NCM460 cells, **, *P* < 0.01. (**C**) Protein expression of CNPY2 isoform2 in CRC cell lines (SW480, DLD-1, SW620, and HT29) and NCM460 cells determined by western blot. (**D**) The relative expression of CNPY2 isoform2 in NCM460, SW480, DLD-1, SW620, and HT29 was 0.41, 0.36, 0.75, 1.22 and 1.38, respectively. (**E**) Expression of the two CNPY2 isoforms was significantly higher in tumor tissues than in tumor-adjacent normal tissues (*n* = 5, **, *P* < 0.01). (**F**) Expression of CNPY2 isoform2 was significantly higher compared to that of CNPY2 isoform1 in tumor tissues (*n* = 5, *, *P* < 0.05). Relative expression of CNPY2 mRNA was normalized to the internal reference gene GAPDH.

CNPY2 isoform1 and isoform2 mRNA was detectable in normal colonic epithelial cells (NCM460) and CRC cells, including SW480, DLD-1, SW620 and HT29, by quantitative real-time polymerase chain reaction (qPCR) analysis. CNPY2 isoform1 was increased in DLD-1, SW620 and HT29 cells, but not in SW480 cells, compared to that in NCM460 cells (Figure 1A, *P* < 0.01). Meanwhile, expression of CNPY2 isoform2 was significantly higher in all CRC cell lines compared to the NCM460 line (Figure 1B, *P* < 0.01). We selected clone 2 of the CNPY2 isoform2 antibody for protein detection as it was the best one for immuno-histochemistry (IHC) (Supplemental Figure 3). We found that CNPY2 isoform2 protein was highly expressed in the CRC cell lines DLD-1, HT29 and SW620 but was weakly expressed in SW480 and NCM460 cells by western blot (Figure 1C and 1D). In addition, we found that the two isoforms of CNPY2 mRNA were significantly increased in 5 CRC tissues compared with the paired tumor-adjacent normal tissues (Figure 1E, *P* < 0.01). Moreover, the expression of CNPY2 isoform2 in CRC tissues was also significantly higher than that of CNPY2 isoform1 (Figure 1F, *P* < 0.05).

### CNPY2 isoform2 expression in CRC tissues

**Figure 2:**
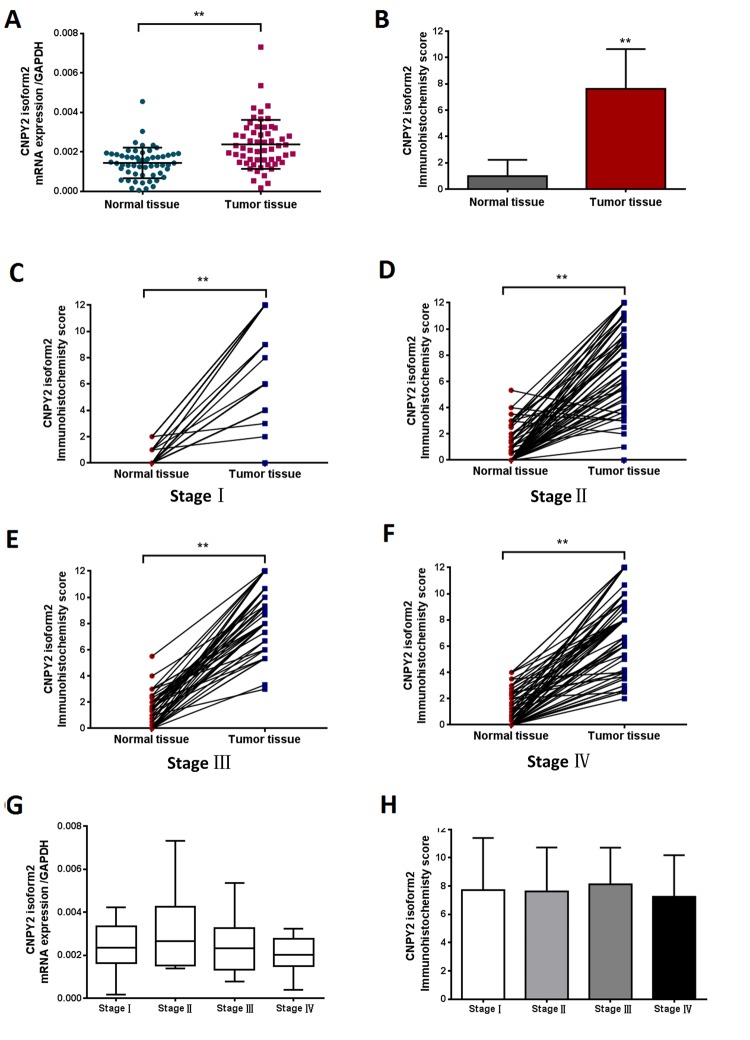
CNPY2 isoform2 expression in CRC tissues detected by RT-PCR and TMA-IHC (**A**) Preferential expression of CNPY2 isoform2 mRNA was observed in CRC tissues compared matched tumor-adjacent normal tissues, (*n* = 57, **, *P* < 0.01). (**B**) Increased expression of CNPY2 isoform2 protein was detected in tumor tissues compared to matched tumor-adjacent normal tissues among all patients (*n* = 285, 7.6 ± 0.2 vs. 1.0 ± 0.1, **, *P* < 0.01). (**C**-**F**) Increased expression of CNPY2 isoform2 protein was detected in tumor tissues compared to matched tumor-adjacent normal tissues among patients with stage I-IV disease (**C**: Stage I,7.7 ± 0.6 vs. 0.5 ± 0.1; **D**: Stage II,7.4 ± 0.3 vs. 1.0 ± 0.2; **E**: Stage III,8.6 ± 0.3 vs. 1.3 ± 0.2; **F**: Stage IV,7.5 ± 0.4 vs. 1.0 ± 0.2; **, all *P* < 0.01). (**G**) Expression level of CNPY2 isoform2 mRNA was slightly higher in stage II CRC but did not reach statistical significance compared to that in other stages (*n* = 57, *P* > 0.05). (**H**) CNPY2 isoform2 protein expression was comparable among patients with disease stage I-IV (*n* =2 85, *P* > 0.05).

**Figure 3:**
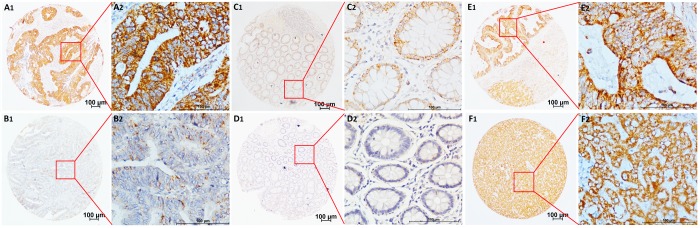
Representative TMA-IHC staining of CNPY2 isoform2 protein (**A1**, **A2**) Colon adenocarcinoma with high CNPY2 isoform2 expression in the cytoplasm of CRC cells and some expression in the extracellular matrix; (**B1**, **B2**) Colon adenocarcinoma with low CNPY2 isoform2 staining in the cytoplasm of CRC cells; (**C1**, **C2**) Tumor-adjacent normal tissues with positive staining of CNPY2 isoform2; (**D1**, **D2**) Tumor-adjacent normal tissues with negative staining of CNPY2 isoform2. (**E1**, **E2**) High CNPY2 isoform2 expression in liver metastatic tumor tissues; (**F1**, **F2)** High CNPY2 isoform2 expression in normal liver tissues. Original magnification was 40×with 100 μm scale per in A1, B1, C1, D1, E1, and F1; 400× with 100 μm scale per bar in A2, B2, C2, D2, E2, and F2.

The CNPY2 isoform2 mRNA levels in the series of 57 tumor tissues and tumor-adjacent normal tissues were further examined in this study. Similar to the results shown in Figure 1E, CNPY2 isoform2 mRNA level was significantly elevated in CRC tumor tissues compared to that in tumor-adjacent normal tissues by qPCR analysis (Figure 2A, *P* < 0.01). In the cohort of 285 patients, the mean IHC score of CNPY2 isoform2 protein expression was substantially higher in tumor tissues than that in tumor-adjacent normal tissues (7.6 ± 0.2 vs. 1.0 ± 0.1, *P* < 0.001, Figure 2B). Moreover, increased expression of CNPY2 isoform2 in tumor tissues was observed in all patients with stage I-IV disease compared to that of tumor-adjacent normal tissues (*P* < 0.001, Figure 2C-F), while the expression levels in tumor tissues were comparable among various stages at both the mRNA and protein level (Figure 2G and H). As shown in Figure 3, expression of CNPY2 isoform2 was observed in primary tumors, liver metastatic tumors, tumor-adjacent normal tissues and normal liver tissues. In addition, positive staining of CNPY2 isoform2 protein was predominantly enriched in the cytoplasm of epithelial cells (Figure 3), while some protein was detected in the extracellular matrix (Figure 3A2).

### Cutoff point for CNPY2 isoform2 expression and clinicopathological characteristics

**Figure 4:**
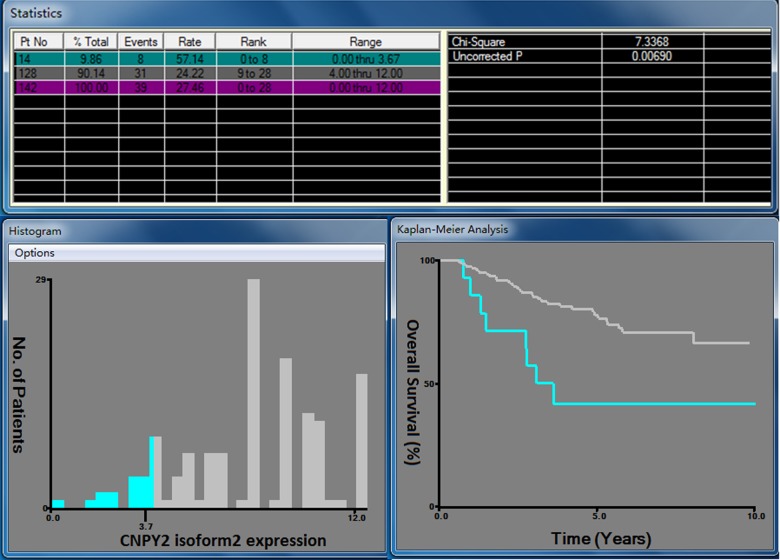
X-tile analysis for OS based on CNPY2 isoform2 expression among patients in the training cohort The optimal cutoff value of the CNPY2 isoform2 expression score was 3.7 at the maximum chi-square value of 7.337.

The 285 cases were randomly divided into a training cohort (n = 142) and an independent validation cohort (n = 143). We selected the optimal cutoff CNPY2 isoform2 score as 3.7 at the highest chi-square value of 7.337 in the training cohort (Figure 4). Patients were further divided into 2 groups: 247 (86.7%) patients in the high CNPY2 isoform2 expression group (IHC score ≥ 3.7) and 38 (13.3%) patients in the low CNPY2 isoform2 expression group (IHC score < 3.7). The associations between CNPY2 isoform2 expression and clinicopathological parameters, including gender, age, tumor location, tumor size, histological type, clinical stage, preoperative serum carcinoembryonic antigen (CEA) and CA199, were assessed. No significant association was found between CNPY2 isoform2 expression with all the above clinicopathological characteristics in total cohorts, the training cohort and the validation cohort (Table 1).

**Table 1:** Relationship between CNPY2 isoform2 expression and clinicopathological characteristics in colorectal cancer patients as shown by immunohistochemical detection

	Total Cohort (n=285, %)			Training Cohort (n=142, %)			Validation Cohort (n=143, %)		
Variable	CNPY2 isoform2 ^Low^	CNPY2 isoform2 ^High^	*P* value	CNPY2 isoform2 ^Low^	CNPY2 isoform2 ^High^	*P* value	CNPY2 isoform2 ^Low^	CNPY2 isoform2 ^High^	*P* value
**Total**	38(13.3)	247(86.7)		14(9.9)	128(90.1)		24(16.8)	119(83.2)	
**Gender**			0.340			0.602			0.514
**Male**	26(14.9)	149(85.1)		9(11.0)	73(89.0)		17(18.3)	76(81.7)	
**Female**	12(10.9)	98(89.1)		5(8.3)	55(91.7)		7(14.3)	43(86.0)	
**Age (year)**			0.745			0.651			0.910
**≤60**	20(14.0)	123(86.0)		8(11.0)	65(89.0)		12(17.1)	58(82.9)	
**>60**	18(12.7)	124(87.3)		6(8.7)	63(91.3)		12(16.4)	61(83.6)	
**Location**			0.404			0.172			0.961
**Colon**	21(12.0)	154(88.0)		6(7.1)	79(92.9)		15(16.7)	75(83.3)	
**Rectum**	17(15.5)	93(84.5)		8(14.0)	49(86.0)		9(17.0)	44(83.0)	
**Tumor size (cm)**			0.500			0.426			0.191
**≤4**	21(14.7)	122(85.3)		5(7.7)	60(92.3)		16(20.5)	62(79.5)	
**>4**	17(12.0)	125(88.0)		9(11.7)	68(88.3)		8(12.3)	57(87.7)	
**Histologic type**			0.133			0.225			0.488
**Well/moderately**	30(12.1)	217(87.9)		10(8.3)	111(91.7)		20(15.9)	106(84.1)	
**Poorly/mucinous**	8(21.1)	30(78.9)		4(19.0)	17(81.0)		4(23.5)	13(76.5)	
**T stage**			0.494			0.053			0.072
**1**	0	12(100)		0	4(100)		0	8(100)	
**2**	10(12.2)	72(87.8)		1(2.4)	40(97.6)		9(22.0)	32(78.0)	
**3**	10(16.1)	52(83.9)		2(5.9)	32(94.1)		8(28.6)	20(71.4)	
**4**	18(14.0)	111(86.7)		11(17.5)	52(82.5)		7(10.6)	59(89.4)	
**N stage**			0.510			0.664			0.255
**0**	19(11.4)	148(88.6)		9(10.3)	78(89.7)		10(12.5)	70(87.5)	
**1**	14(15.9)	74(84.1)		3(7.1)	39(92.9)		11(23.9)	35(76.1)	
**2**	5(16.7)	25(83.3)		2(15.4)	11(84.6)		3(17.6)	14(82.4)	
**TNM stage**			0.498			0.224			0.880
**1**	4(11.4)	31(88.6)		0	17(100)		4(22.2)	14(77.8)	
**2**	12(12.0)	88(88.0)		8(16.0)	42(84.0)		4(8.0)	46(92.0)	
**3**	6(10.0)	54(90.0)		2(6.7)	28(93.3)		4(13.3)	26(86.7)	
**4**	16(17.8)	74(82.2)		4(8.9)	41(91.1)		12(26.7)	33(73.3)	
**Preoperative CEA, ng/ml**			0.496			0.916			0.390
**≤5**	18(12.2)	129(87.8)		8(10.5)	68(89.5)		10(14.1)	61(85.9)	
**>5**	20(15.0)	113(85.0)		6(9.2)	59(90.8)		14(20.6)	54(79.4)	
**Unknown**	0	5		0	1		0	4	
**Preoperative CA199, U/ml**			0.524			0.762			0.227
**≤35**	29(13.1)	193(86.9)		12(10.7)	100(89.3)		17(15.5)	93(84.5)	
**>35**	9(16.4)	46(83.6)		2(7.1)	26(92.9)		7(25.9)	20(74.1)	
**Unknown**	0	8		0	2		0	6	

Abbreviations: TNM: tumor-node-metastasis, CEA: carcinoembryonic antigen

### Association between the CNPY2 isoform2 expression and survival outcome

**Figure 5:**
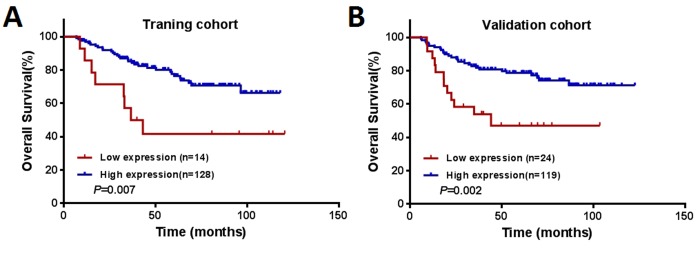
Kaplan–Meier curves of OS among patients with colorectal cancer (**A**) The training cohort (*n* = 142). (**B**) The validation cohort (*n* = 143).

The median follow-up period for all the patients was 58 months (range, 6 – 123 months). During the follow-up period, 71 (24.9%) patients died of disease progression. In the training cohort, low expression of CNPY2 isoform2 was associated with a lower 5-year overall survival (OS) rate compared to that with high expression of CNPY2 isoform2 (41.7% vs. 77.7%, *P* = 0.007, Figure 5A). Low expression of CNPY2 isoform2 also identified patients with a lower 5-year OS rate in the validation cohort (47.1% vs. 78.8%, *P* = 0.002, Figure 5B). As shown in Table 2, univariate analysis revealed that low CNPY2 isoform2 expression was a predictor of worse 5-year OS in both the training cohort [hazard ratio (HR) = 2.809; 95% confidence interval (CI), 1.288–6.125; *P* = 0.009) and validation cohort (HR = 2.885; 95% CI, 1.442–5.654; *P* = 0.003). In the multivariate Cox model, low CNPY2 isoform2 expression was identified as a predictor for shorter 5-year OS in the training cohort (HR = 5.001; 95% CI, 2.156–11.598; *P* < 0.001) and validated in the validation cohort (HR= 2.443; 95% CI 1.197- 4.983, *P* = 0.014). In addition, metastatic disease (stage IV) was a predictor of worse 5-year OS for CRC patients in both the training cohort and validation cohort.

**Table 2:** Univariate and multivariate analysis of clinicopathological factors for 5-year overall survival of colorectal cancer patients with immunohistochemical examination in the training and validation cohorts

	Training cohort						Validation cohort					
	Univariate analysis			Multivariate analysis			Univariate analysis			Multivariate analysis		
Variable	HR	95% CI	*P* value	HR	95% CI	*P* value	HR	95% CI	*P* value	HR	95% CI	*P* value
Gender (Male vs. Female)	0.865	0.461-1.625	0.653				1.343	0.682-2.643	0.394			
Age (> 60 years vs.≤ 60 years)	1.416	0.751-2.667	0.282				1.093	0.587-2.033	0.780			
Location (Rectum vs. Colon)	0.787	0.412-1.502	0.467				0.585	0.297-1.153	0.121			
Tumor size (> 4 cm vs. ≤ 4 cm)	0.785	0.419-1.471	0.450				0.568	0.296-1.091	0.090			
Histologic type (Poor + mucinous vs. Well + moderate)	0.736	0.288-1.885	0.736				1.115	0.437-2.846	0.820			
T stage (3+4 vs. 1+2)	3.501	1.369-8.952	0.009				7.407	2.283-24.032	0.001			
N stage (1+2 vs. 0)	1.436	0.765-2.696	0.260				3.217	1.659-6.241	0.001			
TNM stage (IV vs. I+II+III)	3.399	1.791-6.450	<0.001	3.849	1.975-7.504	<0.001	7.524	3.849-14.710	<0.001	6.480	3.285-12.785	<0.001
Preoperative CEA (> 5 ng/mL vs. ≤ 5 ng/ mL)	2.116	1.110-4.035	0.023				3.488	1.699-7.160	0.001	3.280	1.583-6.796	0.001
Preoperative CA199 (> 35 U/mL vs. ≤ 35 U/mL)	2.664	1.383-5.133	0.003	3.252	1.625-6.509	0.001	2.352	1.185-4.666	0.014			
CNPY2 isoform2 (Low expression vs. High expression)	2.809	1.288-6.125	0.009	5.001	2.156-11.598	<0.001	2.885	1.442-5.654	0.003	2.443	1.197-4.983	0.014

Abbreviations: HR: hazard ratio, CI: confidence interval, TNM: tumor-node-metastasis, CEA: carcinoembryonic antigen

## DISCUSSION

The multiple biologic functions of CNPY2 have been preliminarily elucidated in previous studies. CNPY2 was first revealed as a vital modulator that enhances neurite outgrowth in neuroblastoma and PC12 cells and pro-motes cell migration in rat C6 glioma cells by phospho-rylating and preventing ubiquitination of myosin regula-tory light chain (MRLC) [13,14]. In addition, CNPY2 also participated in lipid metabolism and served as a crucial target for FGF21 to stabilize low-density lipoprotein receptor (LDLR) levels in the mouse macrophage Raw 264.7 cells [15]. In cardiovascular research, CNPY2 was confirmed as a HIF-1a-regulated, secreted angio-genic growth factor, participating in revascularization and subsequently attenuating the transition from compen-satory hypertrophic response to dilated heart failure [12,16]. CNPY2 promoted CRC progression by en-hancing cell proliferation, migration, and angiogenesis and inhibiting apoptosis through upregulation of the p53 pathway [17]. Consistent with these results, CNPY2 also increased renal cancer cell growth by regulating TP53 gene expression [18]. Nonetheless, all the above conclusions were drawn from CNPY2 isoform1.

After searching the GenBank database, we identified CNPY2 isoform2, but it was previously unknown whether it is expressed in human cells or tissues. Although CNPY2 isoform2 had the same first 69 AAs as isoform1, the unique C-terminus of isoform2 (TVTVPPNKVAHSGFG), which replaced P YARSEA-HLTELLEE with no AA 85-182 and the ER retention sequence (HDEL) compared to isoform1, might contribute to distinctive biological functions. For the first time, we showed that expression of CNPY2 isoform2 was significantly higher in CRC cell lines and tumor tissues than that in normal colonic epithelial cells and tumor-adjacent normal tissues, which is consistent with data on CNPY2 isoform1 (Figure 1). In survival analysis, we found that lower CNPY2 isoform2 expression was associated with worse 5-year OS and further showed that it was a predictor for 5-year OS.

CNPY2 isoform1 has been identified as a widespread protein in multiple epithelial tissues, including skin and sebaceous gland, the respiratory system, endocrine glands, the digestive and urinary system, the eye and the reproductive system, particularly in the luminal side of the tissue [11]. Moreover, CNPY2 isoform1 expression was significantly increased in CRC tissues compared with tumor-adjacent normal tissues [17]. The expression of CNPY2 isoform2 was commonly detected in primary CRC tumors, liver metastatic lesions, tumor-adjacent normal tissues and normal liver tissues (Figure 3). Accumulating evidence has shown that CNPY2 isoform1 is a secreted protein and has an extracellular function [11,12,16,17]. Similar to CNPY2 isoform1, CNPY2 isoform2 also has a signal peptide consisting of the first 20 AAs, which was predicted by SignalP 4.1 (http://www.cbs.dtu.dk/services/SignalP/, Supplemental Figure 4A). Furthermore, the remaining AA sequence of CNPY2 isoform2 does not contain any trans-membrane domains predicted by TMHMM Server v. 2.0 (http://www.cbs.dtu.dk/services/TMHMM/, Supplemental Figure 4B), suggesting that it can be processed in the extracellular environment. Further analysis showed that CNPY2 isoform2 was pre-dominantly located in the cytoplasm of epithelial cells, as well as in the extracellular matrix (Figure 3A2). Therefore, we proposed that CNPY2 isoform2 functions as a secreted protein.

Our study also focused on the clinical significance of CNPY2 isoform2 expression on CRC tissues. In the current study, expression of CNPY2 isoform2 was not associated with general clinicopathological variables, including CEA and CA199. Nevertheless, the data suggested that lower CNPY2 isoform2 expression was associated with worse oncologic outcome for CRC patients. Multivariate prognostic analysis identified low CNPY2 isoform2 expression as an unfavorable factor for OS, which was further confirmed in the validation cohort. In contrast to isoform1, which was confirmed to enhance CRC progression [17], we hypothesized that CNPY2 isoform2 acted as a suppressor of CRC metastasis. In this process, Saposin B-like domain of CNPY2 isoform2 might play an important role, due to its function of invoking immunological responses. As a lipid transfer protein, Saposin B could facilitate Natural Killer T (NKT) cell activation through mediating lipids binding to CD1d [19,20]. Furthermore, Saposin B also facilitated antigen cross-presentation by disintegrating membranes of apoptotic vesicles in recipient dendritic cells (DCs), resulting in activated subsequent CD8+ T cell responses [21]. It was clear that both NKT and CD8+ T cells acted as a direct killer to eliminate tumor cells and impede tumor progression [22,23]. Therefore, patients with lower CNPY2 isoform2 expression might have weaker immunological responses to conquer tumor progression, which could translate into a poorer survival outcome. However, the molecular mechanism underlying the effect on the clinical outcomes remains unclear and should be investigated in further studies on CNPY2 isoform2 in tumor development. Nevertheless, CNPY2 isoform2 can be applied in clinical practice and act as a supplementary diagnostic tool for patients. Determining the expression of CNPY2 isoform2 could provide useful information for potential therapeutic choices for patients. For patients with low CNPY2 isoform2 expression, more intensive post-operative chemotherapy and normative follow-up should be performed. Otherwise, low-risk patients with high CNPY2 isoform2 expression may avoid unnecessary post-operative examination and treatment.

Some potential limitations of current study need to be considered. Our study only focused on the expression of CNPY2 isoform2 on CRC cells and tissues, and its expression in other cell lines and tissues is unknown.

The systematic human tissue detection of CNPY2 isoform2 could provide more comprehensive infor-mation on the protein. Since CNPY2 isoform1 was identified as a promoter of CRC growth and development, the prognostic value was not evaluated and compared to that of CNPY2 isoform2. These data will help to determine which of the two isoforms is better for clinical application for diagnosis and monitor-ing CRC patients. As mentioned above, although CNPY2 isoform2 is believed to be a potential secreted protein, this was not validated in the current study. Thus, further investigation is needed to confirm the role of extracellular CNPY2 and reveal its biological functions.

## CONCLUSION

CNPY2 is more abundantly expressed in CRC cell lines and tumor tissues compared to normal colonic epithelial cells and tumor-adjacent normal tissues. For the first time, the current study demonstrated that CNPY2 isoform2 is a novel prognostic predictor for CRC patients. The current study preliminarily revealed the expression in CRC tissues and cell lines, and the biological functions and the clinical value of CNPY2 isoform2 should be explored in the future.

## MATERIALS AND METHODS

### Clinical specimen collection

Paraffin-embedded specimens were collected from 299 patients diagnosed with colorectal adenocarcinoma who underwent primary tumor resection and recruited at Sun Yat-sen University Cancer Center between February 2006 and January 2014. Patients with preoperative anti-cancer treatment, multiple primary CRCs, or a history of other active malignancies (except for basal cell carcinoma of the skin) and those who died or were lost to follow-up within 6 months after surgery were excluded. A total of 285 eligible cases were finally included in current study. Additionally, fresh CRC cancer tissues and the matched tumor-adjacent normal tissues were harvested from an independent cohort of 57 preoperative treatment naive CRC patients and stored at 80°C for biochemical studies. Basic characteristics of the 57 patients are shown in Supplemental Table 1. The detailed clinicopathologic information of the eligible patients was reviewed through the electronic medical records system, and follow-up data were collected from the tracking system. This study was undertaken in accordance with the ethical standards of the World Medical Association Declaration of Helsinki. The study and consent procedure were approved by the Institutional Research Ethics Committee of Sun Yat-sen University Cancer Center (Approval number: B2017-042-01), and informed consents for using tissue samples, before the initial treatment, were obtained from the patients.

### Cell lines and culture

Human CRC cell lines (SW480, SW620, HT29 and DLD-1) were purchased from the American Type Culture Collection (ATCC, Manassas, VA, USA), while the human normal colon epithelial cell line NCM460 was acquired from INCELL (San Antonio, TX, USA). Before use, all cells were negatively tested for myco-plasma contamination and authenticated based on STR fingerprinting at Medicine Lab of Forensic Medicine Department of Sun Yat-sen University. HT29 was cultured in McCoy's 5A medium, while the remaining cell lines were cultured with RPMI-1640 medium (Gibco, Grand Island, NY, USA); both were supplemented with 10% fetal bovine serum (FBS, Gibco Invitrogen Co., Grand Island, NY, USA), 100 units/ml penicillin and 100 mg/ml streptomycin. All cell lines were maintained at 37°C in a humidified atmosphere of 5% CO_2_.

### Generation of CNPY2 isoform2 monoclonal antibodies

The open reading frame of CNPY2 isoform2 (GenBank accession. no. NM_001190991) encoding amino acids (AAs) 21–84 (excluding the first 20 AAs as the predicted signal peptide) was polymerase chain reaction (PCR)-amplified from the total cDNA of human smooth muscle cells and inserted into the pET30a vector between HindIII and XhoI restriction sites, resulting in recombinant CNPY2 fused to a 6×His tag at its C-terminus (CNPY2-His). The *E. coli* BL21 (DE3) strain was transformed with recombinant plasmids. A single colony was inoculated into medium containing the appropriate antibiotic (kanamycin). Isopropyl beta-D-1-thiogalactopyranoside (IPTG) was introduced for induction, and CNPY2 isoform2-His recombinant protein was purified from inclusion bodies. Proteins were analyzed by 15% sodium dodecyl sulfate-poly-acrylamide gel electrophoresis (SDS-PAGE) and western blot using standard protocols for molecular weight and purity measurements. The theoretical isoelectric point of this recombinant CNPY2 isoform2-His protein is ~7.27, and the theoretical molecular weight is ~7.96 kDa (DNA and protein sequences are shown in Supplemental Table 2). This recombinant CNPY2 isoform2-His protein was used to immunize mice for four rounds of injection, including booster immunization, and eventually, the splenocytes were fused with myeloma cells to generate hybridoma clones. Then, 5 positive clones were selected by enzyme-linked immunosorbent assay (ELISA) using the short peptide as the mapping epitope. We compared the efficacy of these 5 clones using IHC, and finally, we selected the best clone for IHC staining. The selected clone antibody binds to the epitope that was unique to the CNPY2 isoform2 C-terminus region and does not cross react with isoform1 or any other known proteins. Monoclonal antibody was purified from the supernatant of the selected clone using a standard protein A/G protocol.

### Detection of CNPY2 mRNA

The total cellular RNA was extracted from frozen samples and cultured cells using TRIzol reagent (Invitrogen, CA, USA), and DNase I was applied to exclude potential genomic DNA contamination. Reverse transcription (RT) was performed using a Revert Aid First Strand cDNA Synthesis Kit (Thermo Scientific, Waltham, MA, USA) following the manufacturer's instruction. The RT reaction was incubated at 25°C for 5 min, then at 42°C for 60 min and terminated at 70°C for 5 min. qPCR primers were designed and are presented in Supplemental Table 3. The primers designed for the CNPY2 isoform2 only amplified CNPY2 isoform2 and not isoform1 or any other known transcripts, which was confirmed in human smooth muscle cells (Supplemental Figure 2). Real-time PCR amplification was performed with DreamTaq DNA Polymerase (Thermo Scientific, Waltham, MA, USA) using the ROCHE 480 system. First, the reaction was performed at 95°C for 5 min with an S1000 Thermal Cycler (Bio-Rad) for initial denaturation, and then, amplification was performed at 94°C for 30 s, 60°C for 30 s, and 72°C for 30 s for 40 cycles, followed by a final extension at 72°C for 7 min. Relative quantification of CNPY2 mRNA was performed using the 2^−ΔΔCt^ method.

### Western blot analysis

Western blot analysis was conducted following our previously described method [24]. Briefly, equal amounts of protein were loaded and separated using 15% SDS-PAGE. The proteins were then electro-phoretically transferred onto a polyvinylidene fluoride (PVDF) membrane. The membrane was incubated with the CNPY2 isoform2 and β-actin antibody (1:5000 dilution, 4970S, Cell Signaling Technologies, Danvers, MA, USA) at 4°C overnight. The membrane was washed and incubated with a secondary antibody at room temperature for 1 hour. After the membrane was washed 3 times with Tween (TBS-T) buffer, the proteins were detected using an enhanced chemi-luminescence (ECL) reagent kit.

### Tissue microarrays (TMAs) and IHC

TMA slides contained 285 CRC and tumor-adjacent normal tissues, including 48 cases with liver metastasis specimens. TMAs were constructed using a tissue array instrument (Beecher Instruments, Sun Prairie, WI, USA). Briefly, each tissue core with a diameter of 0.6 mm was punched from the marked areas in selected FFPE tissues and organized in the TMA blocks. The blocks were sectioned into 4 μm slices and then mounted onto glass slides. After dewaxing and treat-ment with 0.3% hydrogen peroxide, the slides were incubated with a primary CNPY2 isoform2 antibody (1:800 dilution) overnight in a moist chamber at 4°C. Subsequently, the slides were incubated with anti-mouse/rabbit secondary antibody from REAL En Vision (Dako, Glostrup, Denmark) for 30 min at 37.5°C. The IHC staining was followed by 3′, 3-diaminobenzidine tetrahydrochloride (DAB, Dako, Glostrup, Denmark) staining.

IHC scoring was determined by the percentage and intensity of positively stained cell. The positive staining was scored as follows: “0” (less than 5% positively stained cells), “1” (5–24% positively stained cells), “2” (25–49% positively stained cells), “3” (50–74% positively stained cells), and “4” (75–100% positively stained cells). The intensity was scored according to the following standard: “0” (negative staining); “1” (weak staining); “2” (moderate staining), and “3” (strong staining). The final score was generated by multiplying the percentage score with the staining intensity score. Two trained pathologists blindly evaluated all the specimens.

### Definition of survival endpoint

Follow-up data of all included patients were available for analysis. Within the follow-up period, OS was defined as the length of time (in months) from the date of surgery until death from any cause or last follow-up. The last follow-up visit was in March 2017.

### Statistical analysis

An optimal cutoff value for the IHC score that separates patients into low and high CNPY2 isoform2 expression groups with respect to OS was determined using X-tile software (version 3.6.1; Yale University, New Haven, CT, USA) at the largest chi-square value in the training cohort, as described previously[25,26]. Categorical variables are presented as percentages, and they were compared using the chi-square test or Fisher's exact test. Continuous variables are presented as the median (range) or mean (standard deviation). Student's *t*-test was used for two-group comparison and one-way ANOVA for multiple-group comparison. Survival outcomes were summarized by the Kaplan–Meier method. Potential effects of clinical variables on OS were examined using univariate Cox's proportional hazards model; variables that were statistically significant in univariate Cox models were further assessed with multivariate Cox models using a forward stepwise method. HRs and CIs were subsequently calculated. All analyses were performed using IBM SPSS statistics software, version 21.0 (IBM Corp., Armonk, NY, USA) and GraphPad Prism version 6.0 (GraphPad Software, La Jolla, CA, USA). *P*-values less than 0.05 were considered significant based on two-sided statistical tests.

### Ethics approval and consent to participate

Study was approved by the Institutional Research Ethics Committee of Sun Yat-sen University Cancer Center (Approval number: B2017-042-01).The informed consents for using tissue samples, before the initial treatment, were obtained from the patients.

### Consent for publication

Not applicable.

### Availability of data and materials

The datasets analysed during the current study were available from the corresponding author on reasonable request. Anyone who is interested in the information should contact fangyj@sysucc.org.cn and wands@sysucc.org.cn.

## SUPPLEMENTARY MATERIAL FIGURES AND TABLES


