# Supplementary material for: Expression of a novel CNPY2 isoform in colorectal cancer and its association with oncologic prognosis
Source: Aging (Albany NY). 2017 Nov 13;9(11):2334–51. doi: 10.18632/aging.101324 (PMC5723690; doi:10.18632/aging.101324)
Supplement: Supplementary file 1 [file aging-09-2334-s001.pdf]

SUPPLEMENTAL MATERIAL

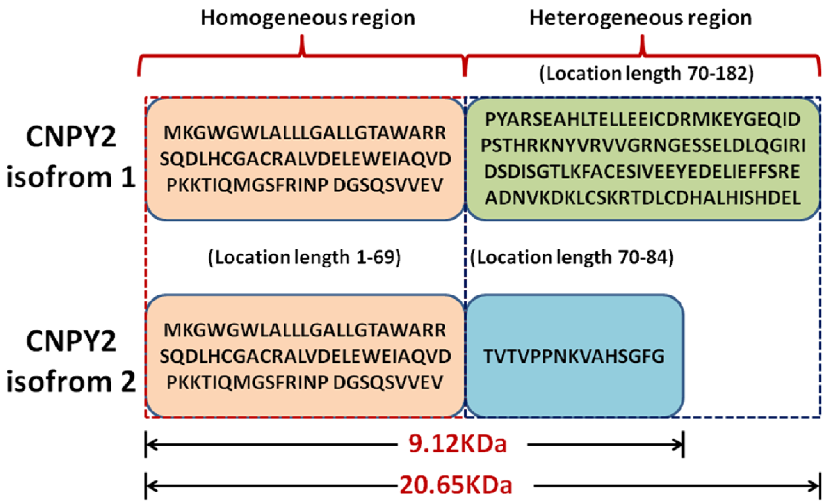

Supplemental Figure 1. Comparison of the protein sequence of the two CNPY2 isoforms. The first 69 AAs is the homogeneous region, while another peptide (TVTVPPNKVAHSGFG) is specific and unique to the C-terminus of CNPY2 isoform2, which is different from CNPY2 isoform1.

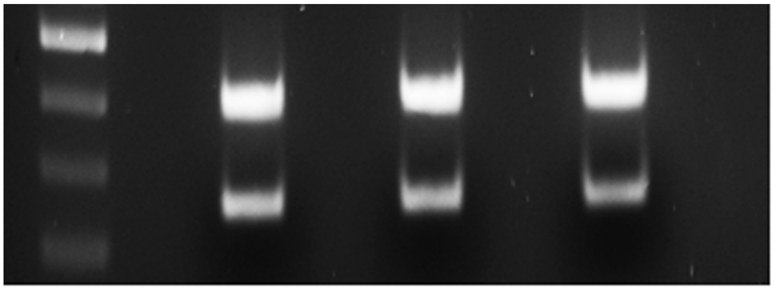

Supplemental Figure 2. Identification of CNPY2 isoform2 with specific primers in human smooth muscle cells using PCR detection. The DNA band at 255 bp is the coding region of CNPY2 isoform2, and the 400 bp band contains intron sequences. The 255 bp DNA band sequence cloned into the PCR2.1 vector was confirmed by sequencing to be the same cDNA sequence as NM_001190991.

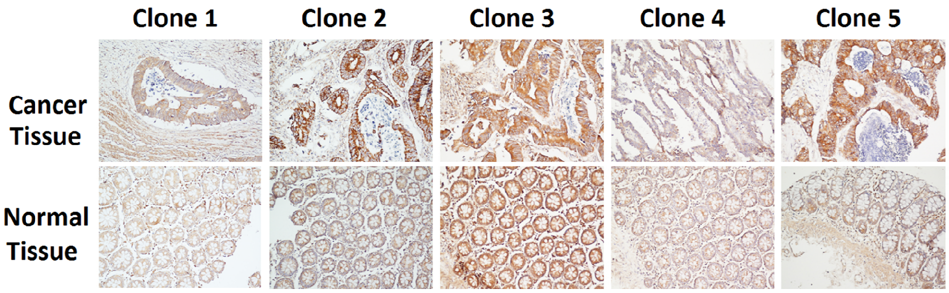

Supplemental Figure 3. Immunohistochemical staining of 5 clones of CNPY2 isoform2 in colorectal cancer tissues. Original magnification is 20× for each picture. Clone 2 is the most specific antibody for targeting epithelial tissues, which was identified as the best one for protein detection.

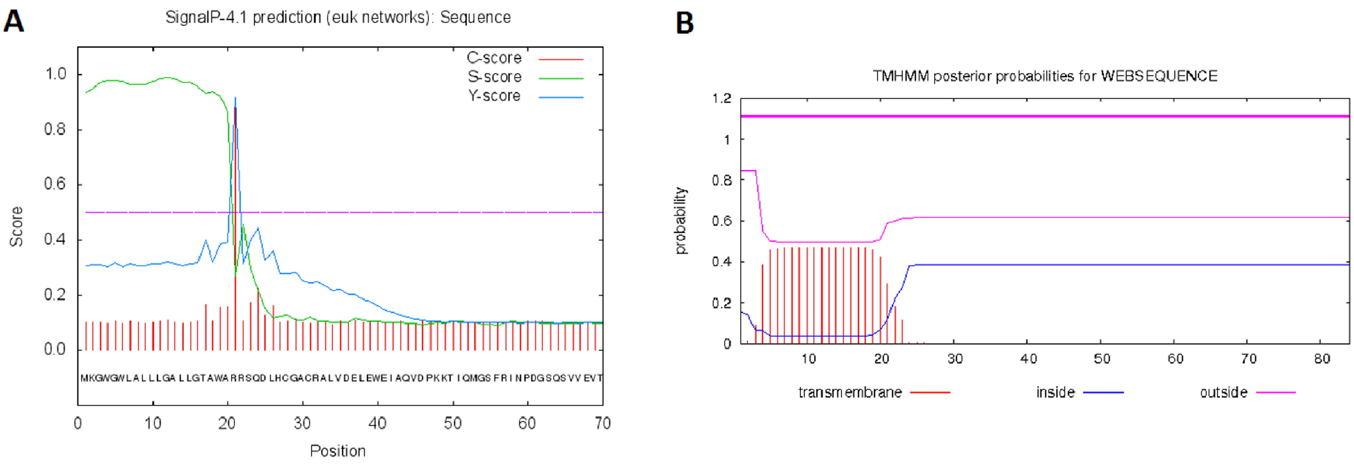

Supplemental Figure 4. Prediction of CNPY2 isoform2 as a secretory protein (**A**) CNPY2 isoform2 contains a signal peptide in the first 21 AAs. The mean S-score of the first 20 AAs is 0.958, and the maximum Y-score at the 21st position AA is 0.918. The discrimination score 0.939 exceeds the cutoff value of 0.450. (**B**) CNPY2 isoform2 does not contain transmembrane domains. The expected number of AAs in transmembrane helices in TMHs is 8.48, which is less than 18; thus, it is unlikely to be a transmembrane protein.

Supplemental Table 1. Clinical characteristics of 57 patients with fresh colorectal cancer tissues for PCR detection.

Characteristics	N = 57 (%)
Age, year (median, range)	59(30-75)
Gender	
Male	35(61.4)
Female	22(38.6)
Location	
Colon	28(49.1)
Rectum	29(50.9)
T stage	
2	13(22.8)
3	20(35.1)
4	24(42.1)
N stage	
0	25(43.9)
1	28(49.1)
2	4(7.0)
TNM stage	
Ⅰ	10(17.5)
Ⅱ	8(14.0)
Ⅲ	19(33.3)
Ⅳ	20(35.1)

Abbreviations: PCR: polymerase chain reaction, TNM: tumor-node-metastasis

Supplemental Table 2. DNA and protein sequence of CNPY2 isoform2.

Location	Sequence
	DNA
1	CGCCGTAGCC AGGATCTGCA TTGCGGTGCG TGCCGTGCGC TGGTTGACGA ACTGGAGTGG
61	GAAATCGCGC AGGTTGACCC GAAGAAAACC ATCCAGATGG GTAGCTTCCG TATTAACCCG
121	GACGGCAGCC AAAGCGTTGT TGAGGTTACC GTGACCGTTC CGCCGAATAA AGTTGCGCAT
181	AGCGGTTTTG GC
	Protein
1	MRRSQDLHCG ACRALVDELE WEIAQVDPKK TIQMGSFRIN PDGSQSVVEV TVTVPPNKVA
61	HSGFG**HHHHHH**

Supplemental Table 3. The designed quantitative PCR primers in the current study.

Biomarker	Direction	Primer sequences
CNPY2 isoform1	Forward	5'-AGACCATTCAGATGGGATCTTTC-3'
	Reverse	5'-CTCCTTCATCCGGTCACATATC-3'
CNPY2 isoform2	Forward	5'-AGACCATTCAGATGGGATCTTTC-3'
	Reverse	5'-TTCATCCAAAGCCAGAGTGAG-3'
GADPH	Forward	5'-TGACTTCAACAGCGACACCCA-3'
	Reverse	5'-CACCCTGTTGCTGTAGCCAAA-3'

Abbreviations: PCR: polymerase chain reaction
